# Parent-child interactions and objectively measured child physical activity: a cross-sectional study

**DOI:** 10.1186/1479-5868-7-71

**Published:** 2010-10-07

**Authors:** Erin Hennessy, Sheryl O Hughes, Jeanne P Goldberg, Raymond R Hyatt, Christina D Economos

**Affiliations:** 1 Cancer Prevention Fellowship Program, National Cancer Institute, National Institutes of Health, Bethesda, Maryland, USA; 2 Department of Pediatrics, Baylor College of Medicine, USDA/ARS Children's Nutrition Research Center, Houston, Texas, USA; 3 Tufts University Friedman School of Nutrition Science and Policy, Boston, Massachusetts, USA; 4 Department of Public Health and Community Medicine, Tufts University School of Medicine, Boston, Massachusetts, USA; 5 John Hancock Research Center on Physical Activity, Nutrition, and Obesity Prevention, Tufts University Friedman School of Nutrition Science and Policy, Boston, Massachusetts, USA

## Abstract

**Background:**

Parents influence their children's behaviors directly through specific parenting practices and indirectly through their parenting style. Some practices such as logistical and emotional support have been shown to be positively associated with child physical activity (PA) levels, while for others (e.g. monitoring) the relationship is not clear. The objectives of this study were to determine the relationship between parent's PA-related practices, general parenting style, and children's PA level.

**Methods:**

During the spring of 2007 a diverse group of 99 parent-child dyads (29% White, 49% Black, 22% Hispanic; 89% mothers) living in low-income rural areas of the US participated in a cross-sectional study. Using validated questionnaires, parents self-reported their parenting style (authoritative, authoritarian, permissive, and uninvolved) and activity-related parenting practices. Height and weight were measured for each dyad and parents reported demographic information. Child PA was measured objectively through accelerometers and expressed as absolute counts and minutes engaged in intensity-specific activity.

**Results:**

Seventy-six children had valid accelerometer data. Children engaged in 113.4 ± 37.0 min. of moderate-vigorous physical activity (MVPA) per day. Children of *permissive *parents accumulated more minutes of MVPA than those of *uninvolved *parents (127.5 vs. 97.1, *p *< 0.05), while parents who provided above average levels of support had children who participated in more minutes of MVPA (114.2 vs. 98.3, *p *= 0.03). While controlling for known covariates, an *uninvolved *parenting style was the only parenting behavior associated with child physical activity. Parenting style moderated the association between two parenting practices - reinforcement and monitoring - and child physical activity. Specifically, post-hoc analyses revealed that for the *permissive *parenting style group, higher levels of parental reinforcement or monitoring were associated with higher levels of child physical activity.

**Conclusions:**

This work extends the current literature by demonstrating the potential moderating role of parenting style on the relationship between activity-related parenting practices and children's objectively measured physical activity, while controlling for known covariates. Future studies in this area are warranted and, if confirmed, may help to identify the mechanism by which parents influence their child's physical activity behavior.

## Background

Increasing the proportion of children who engage in regular physical activity continues to be a public health priority. Physical activity provides important health benefits for children including increased physical fitness, reduced body fatness, favorable cardiovascular and metabolic disease risk profiles, enhanced bone health, and reduced symptoms of depression and anxiety [1]. Despite the known benefits, few US children meet the current recommendation of 60 minutes of physical activity per day [2], and unless they participate in a structured sport children are likely to become more sedentary as they age [1,3,4]. The problem of physical inactivity may be even more severe in rural areas of the US. Rural children often do not participate in after school sports due in part to limited opportunities and transportation barriers [5]. This may contribute to a greater prevalence of leisure-time inactivity and obesity among rural residents as compared to their metropolitan and suburban counterparts [3,4].

Understanding the factors that influence child physical activity habits is important if we are to try to close the gap between current health behaviors and national recommendations. Health-related behaviors and patterns are established during childhood and adolescence, and evolve within the context of the family [6]. Although parents represent only one possible area of influence (e.g. peers, school), socialization of many behaviors occurs within the family, with parents' beliefs, attitudes, and behaviors substantially affecting children's health [7].

Parents, in particular, exert their influence on their children directly through specific parenting practices and indirectly through their parenting style [8]. Parenting practices are the behavioral strategies that parents employ to socialize their children. In the context of physical activity, practices such as logistical and emotional support and direct modeling have been shown to be positively associated with higher child physical activity levels [9-13]. Other parenting strategies that may positively impact children's physical activity include incorporating activity into family recreational routines, making activity-related equipment available at home, identifying safe places in the community that children can easily access, and finding activities to do outdoors for all weather conditions [14].

In 2000, Sallis et al. concluded that parental influence over child physical activity was indeterminate [15]. A more recent review by van der Horst and colleagues [16] found that several factors were positively associated with child physical activity including gender (male), self-efficacy, parental physical activity (for boys), and parent support. In a meta-analytic review, Pugliese and Tinsley [7] also found that a moderate positive relationship exists between parental support and modeling behavior and child physical activity levels. In fact, children had a relative risk of being inactive that was 1.41 times greater if parents did not engage in certain socialization behaviors (encouragement, instrumental, and modeling behaviors) than when they did engage in those behaviors. Other parental behaviors may also influence child physical activity. In a study of 800 Latino parents and their children, Arredondo et al. [17] found that parental reinforcement and monitoring were both positively associated with child physical activity. However, the authors noted that, in general, these broader aspects of parenting behaviors toward child physical activity remain understudied, perhaps due to a paucity of measurement tools to assess these constructs [18].

Certain parental practices may be especially important for particular groups of children. Compared to urban areas, rural communities face higher obesity rates for children, and lower physical activity levels may contribute to this difference. Oleson and colleagues [19] found that the distance between homes and physical amenities is great in rural areas, which suggests that children are dependent on their parent (or caregiver) for transportation to places to be physically active. Rural areas also tend to lack sidewalks and other amenities that would support recreational or leisure time activity [20]. In general, there are few rural-based studies in youth suggesting that this population requires more attention and study.

It is also important to note that parenting practices are related to, but distinct from parenting style. According to Darling and Steinberg's conceptual model [21], parenting style is theoretically independent of specific socialization content and influences child development indirectly by changing the effectiveness of the parenting practice. It is based on the idea that parents' attitudes and the beliefs they hold about how they should rear their children result in a two-way interaction that defines the emotional climate of the parenting environment. This dynamic process alters how children view their parents and thus changes how receptive children are to their parents' socialization demands. In other words, parenting style can either undermine or facilitate the parenting practices a parent employs to socialize his or her child.

This model is based on previous work by Baumrind [22] who defined parenting style using two dimensions of parental behavior: responsiveness or nurturance to and demandingness or control of the child. *Responsiveness/nurturance *was defined as "the extent to which parents foster individuality and self-assertion by being attuned, supportive, and acquiescent to children's requests" while *demandingness/control *refers to "claims that parents make on children to become integrated into society through behavior regulation, direct confrontation, maturity demands, and supervision of children's activities." A parent with an *authoritarian *style (high demandingness, low responsiveness) attempts to control their child's behavior with little regard for the child's needs and strict obedience to the parent while an *authoritative *parent (high demandingness, high responsiveness) provides encouragement for the child to express independence, clear set of boundaries, and open communication. A *permissive *parenting style (low demandingness, high responsiveness) expresses greater parental acceptance toward the child, but provides few boundaries and places few demands on the child and, lastly, an *uninvolved *parenting style (low demandingness, low responsiveness) offers few parental boundaries and little interaction with the child.

In general, evidence supports the association between *authoritative *parenting and positive child health outcomes across multiple domains [23-25]. Within the realm of physical activity, few studies have examined the role of parenting style in shaping children's activity-related behaviors. One study by Schmitz et al. [26] found that *authoritative *parents have children who report higher levels of physical activity. A more recent longitudinal study of parental influences on adolescent physical activity showed that factors such as family cohesion, parent-child communication, and parental engagement positively predicted moderate-to-vigorous physical activity for boys and girls after one year [27]. The authors suggest that these parenting behaviors may be reflective of an *authoritative *parenting style in that they provide support for adolescent physical activity and are balanced by appropriate levels of autonomy. Yet, the majority of studies looking at parental influence on child physical activity have either not measured parenting style or not examined this construct in a systematic way. Knowing that parenting style has an impact on multiple domains and appears to be related to child physical activity, more work is needed in this area to better understand the parent-child relationship in the context of physical activity.

**Figure 1:**
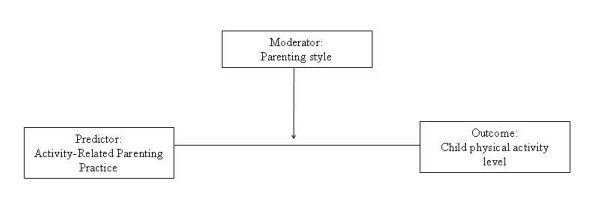
**Moderating role of parenting style in the parent-child physical activity relationship**. Adapted from Darling and Steinberg's conceptual model [21]of parenting style.

To fill this research gap, the current study explores the relationship between parenting styles and practices, and children's physical activity levels. The first aim was to understand the association between general parenting styles and activity-related practices of rural US parents. It has been argued that if practices are independent of a parent's style, then certain practices could be targeted singly while those that are linked require interventions that treat underlying family dynamics as a whole [28]. The second aim was to evaluate the relationship between parenting style, parenting practices, and child activity while controlling for known covariates. A weakness noted in the literature has been the reliance on self-reported activity; therefore, we used an objective measure - accelerometry - to capture children's activity level. In addition, we examined other variables including child weight, which may be related to children's physical activity. Finally, following the theoretical framework described by Darling and Steinberg [21], we tested the moderating role of parenting style on the relationship between activity-related practices and children's physical activity (Figure 1).

## Methods

### Study Setting

Tufts University and Save the Children, U.S. Programs - a non-profit organization that collaborates with schools in under-resourced rural communities to implement early childhood development, literacy, and physical activity and nutrition programs - conducted this observational study. The study sampling frame was developed from Save the Children's U.S. Programs' rural partner schools (children in grades K-5) and categorized by geographic location: Central Valley of California (n = 11 schools), Mississippi River Delta (n = 15 schools), Southeastern region (n = 9 schools) and Appalachian region (n = 48 schools). Rural was defined according to the locale codes developed by the National Center for Education Statistics because they are based on the specific conditions of schools and refer to very small geographic areas and circumstances, such as population density and size, which are most likely to be homogeneous in such small areas [29]. Thus, locale codes generally provide the most accurate type of community where students reside.

One school from each region was randomly selected to participate in the study. Participating schools were located in California (CA), Mississippi (MS), South Carolina (SC), and Kentucky (KY). The average school size was 347 students and 98% (SD ± 4.5) participated in the free- and reduced-price lunch program. The median household income in the communities from which our sample population was drawn ranged from $15,923 to $26,937 US (2000) dollars with an average of $21,536 (versus $48,201 nationally) [30].

After randomly selecting the target schools, we met with the principals to enlist their support. One school declined the invitation and another school from that region was randomly selected to participate. Once the school administrator gave support, recruitment letters and flyers, translated into Spanish where appropriate, were sent home to all 6-11 year old children attending the four schools. Flyers were also posted at each school. Our goal was to recruit children and their parents until we reached our target sample of 80 parent-child dyads (approximately 20 per school). Given the possibility of dropouts, with Institutional Review Board (IRB) approval we over-recruited, resulting in a total sample of 99 parent-child dyads from the four regions. It is not possible to sufficiently describe the response/refusal rate for each community as we accepted the first 20-25 dyads per region.

### Study Sample

We collected cross-sectional data from a multi-ethnic sample (29% White, 49% Black, and 22% Hispanic) of 99 parent-child dyads from April through June 2007. A "parent" was defined as either the biological parent or legal guardian with whom the child lived. Children had to be between the ages of 6-11 years and not follow a special diet for medical reasons. Only one parent and one child per family were included in the study. If a parent had two children within this age range, the parent selected which child would participate in the study.

### Procedures

Measurements were conducted on two days - one week apart - at each of the four schools. The measurements took approximately two hours and were collected during after school time. The order of tests was standardized throughout the study. Food and beverages were provided and children received prizes (e.g. jump ropes) at the end. Participants also received a monetary gift card for completion of the study (a quarter of the payment was distributed after the first visit and the rest after the second visit). Only those children and their parents who signed an informed consent (plus an additional assent form given to children ≥ 7 years of age) participated. This study was reviewed and approved by the Tufts University IRB.

### Questionnaires

Existing parental questionnaires were chosen for this study, in part, based on the literacy level of the instrument (5.6 grade level). They were translated into Spanish. Sixteen parents completed the Spanish version of each self-report measure.

#### Parenting dimensions inventory - short form (PDI-S)

The PDI-S [31,32] is a self-administered instrument that examines five dimensions of parenting: support (nurturance), control (type of control, amount of control), and structure (consistency and organization). The short version of this instrument retains the most reliable and valid components of the original PDI with an internal consistency ranging from 0.66 to 0.92 [31]. Coefficient alphas for this sample ranged from 0.66 to 0.96. Using the typological approach of Maccoby and Martin [33], parenting style was categorized into four types (*authoritative*, *authoritarian*, *permissive*, and *uninvolved*) based on median splits of the nurturance and amount of control subscales.

#### Activity-related parenting practices

Two questionnaires from the published literature were combined to assess parenting practices related to child physical activity. One questionnaire measures logistical support (3 items) and explicit modeling (4 items) [9]. Internal consistency was reported as 0.68 and 0.72, respectively for the original questionnaire, and 0.67 and 0.55 for the current study. Following the procedures described by Davison [9], parental support for children's physical activity was calculated from the logistical support and explicit modeling scales, and further dichotomized to reflect above and below average levels of support. The Parenting Strategies for Eating and Activity Scale (PEAS) [17,18] measures parental discipline (2 items), control (1 item), limit setting (4 items), reinforcement (1 item), and monitoring (2 items) in relation to children's physical activity habits. The internal consistency for the limit setting subscale was 0.87 in the original study and 0.83 for this sample. Evidence of test-retest reliability, convergent validity, and predictive validity for both questionnaires has been previously demonstrated [9,17,18].

#### Sociodemographic information

Parents reported their child's date of birth, gender, and ethnicity, and their own age, gender, race/ethnicity, marital status, education level, family characteristics (number of siblings, number of adults in the home), country of birth and number of years and/or months living in the US.

### Physical Activity

All children were asked to wear an Actigraph (Actigraph, LLC: Pensacola, FL) model 7164 accelerometer over the right hip on an elasticized belt for the time between the first and second visits (average wear length = 6 days, including both weekend days). The pocket-size monitor weighs 6 ounces and has been shown to be an excellent tool for measuring children's activity levels [34-36]. Monitors were calibrated (using the manufacturer's calibrator, model CAL71) and initialized the day before they were distributed. All participants received instruction regarding the appropriate placement of the device and to wear it at all times except while sleeping and during water-based activity (i.e., swimming, bathing, etc.). Monitors were collected at the second measurement visit by field staff.

The uniaxial Actigraph measures and records vertical acceleration as "counts," providing an indication of the intensity of physical activity associated with locomotion. Counts were obtained in 30-second increments known as "epochs". Through a sequence of data reduction steps physical activity variables were created. First, counts were screened for spurious data (e.g. an extended sequence of the maximum recordable value, counts beyond the biologically plausible range, or sequences of 60+ minutes in which activity never returned to zero) [2,37]. Second, a valid day was defined as having at least 80% of a standard day, the length of time in which 70% of the sample wore the monitor. Wear time was determined by subtracting non-wear time from 24 h. Non-wear was defined by an interval of at least 60 consecutive minutes of zero activity intensity counts, with allowance for 1-2 min of counts between 0 and 100 [2,37]. To be included in the analysis, participants had to have four or more valid days, including at least one weekend day.

The amount of physical activity is presented as total counts and estimates of the time spent in physical activity according to count thresholds. Total counts evaluate the raw data provided by the accelerometer without any external criteria other than determination of wear and non-wear time [2]. Time spent in physical activity of varying intensity levels (light, moderate, vigorous, very vigorous) was based on application of count thresholds corresponding to intensity-specific activity. We used the cut points established by Freedson [34] and adapted these for a 30-s epoch. Data was also summed for any activity greater than moderate intensity for a total of moderate-vigorous physical activity (MVPA).

### Anthropometric Measures

Height and weight measurements were obtained on the parent and child, and taken in triplicate following standardized procedures [38]. Height was measured, without shoes, to the nearest eighth of an inch using a portable stadiometer (Shorr Infant/Child/Adult Height/Length Measuring Board; Shorr, Olney, MD). Weight was measured in light clothing to the nearest 0.1 lb on a portable digital scale (Befour PS-6600 Portable Scale; Befour, Inc., Saukville, Wisconsin). Body mass index (BMI) was calculated from the average of the three body weight and height measurements for each dyad. For children, BMI was transformed into z scores using the age- and sex-specific CDC reference standards [39]. The following terminology was utilized to classify child weight categories: underweight (< 5^th ^percentile), normal weight (5^th^-84^th ^percentile), overweight (85^th^-94^th^), and obese (≥ 95^th^) [40]. Parent BMI was classified into the following weight categories: underweight (< 18.5), normal weight (18.5-24.9), overweight (25-29.9), obese (30-39.9), and extreme obesity (≥ 40) [41].

### Data Analysis

Activity-related parenting practice subscales were centered (revised sample mean = 0) due to the different number of response options across items and to allow for more interpretable coefficients [42]. Indicator variables were computed for each parenting style typology. Descriptive statistics were calculated to verify normal distribution. Independent samples t-tests were used to examine the differences between boys and girls and to determine minutes of physical activity by parent support. Generalized linear models were used to determine differences in children's physical activity and parenting style typologies. Spearman rank correlations were used to test for associations between parenting styles and practices, and child physical activity level, while Pearson correlations were used to test for associations between potential covariates and child physical activity.

Multiple linear regression models examined the association between child physical activity and parenting style and activity-related practices independently, while controlling for covariates that were significantly associated with the outcome. Based on previous research and our own hypotheses, regression models were run using an *authoritarian *style as the referent group. Historically, an *authoritative *parenting style has been shown to predict the most positive child outcomes [22,33,43,44]. Results from our work [45] and others [46,47] also suggest that other parenting styles, such as *permissive *and *uninvolved*, may also be associated with child health behaviors, but have not been adequately examined. Therefore, we selected a priori for an authoritarian parenting style to be the referent group in the regression models.

Following standard procedures [48], moderated multiple regression (MMR) analysis was used to examine the effect of the moderator variable (parenting style) on the relation between the dependent variable (total counts or minutes of MVPA) and an independent variable (e.g. a particular parenting practice such as logistical support). To do this, a restricted model comprised of the independent variable and the hypothesized moderator was first created by entering both terms as a block. Next, a full MMR model was constructed by adding the focal interaction term to the restricted model. The interaction term was computed by multiplying each centered practice score with each parenting style typology indicator variable. Covariates that were not significantly associated with the outcome were omitted from the final models. Standard statistical tests were used to determine if the incremental variance (*R*^2 ^change) explained by the interaction terms was significant. A post-hoc analysis to probe for any significant moderation effect was conducted according to the procedures described by Aiken & West [48] and Holmbeck [49]. Statistics conducted with the data were run using SPSS (version 14.0; SPSS Inc., Chicago, IL) and SAS (version 9.1; SAS Institute Inc., Cary, NC). An alpha level of 0.05 was used for all statistical tests. Findings are reported as mean ± standard deviation (SD) unless otherwise noted.

## Results

### Participant profiles

Of the total sample, 76 children had valid accelerometer data. Data from eight monitors were lost in the field while 15 malfunctioned (corrupt batteries resulting in no data being recorded (n = 5) or data with extended sequences of the maximum recordable value (n = 10)). Due to this loss of data, we focused all subsequent analyses on the analytic sample (n = 76) as opposed to the full sample (n = 99). The children who had valid accelerometer data did not differ demographically or by BMI z score from the full study population.

Table 1 shows that children were, on average, 9.05 (± 1.5) years old and mostly female (n = 50, 66%). BMI z score was 1.12 (± 0.9) with 58% of children classified as overweight or obese. The majority of parents were female (n = 73, 96%), the mother of the participating child (n = 65, 89%), and between the ages of 30-39 years (n = 38, 50%) with a high school education or less (n = 38, 50%). Of the participants who self-identified as Hispanic (n = 19, 25%), fourteen (74%) were either Mexican or Mexican-American. Fifteen participants (79%) were born in a country other than the US and the number of years living in the US was 13.04 (± 7.4) years. Half of the parents (50%) were married. Mean parental BMI was 31.88 (± 8.8) corresponding to 24% overweight, 33% obese and 19% extremely obese.

**Table 1:** Demographic characteristics of the n = 76 parent-child dyads living in four underserved US rural communities.

Child	
Gender, *n*	26M, 50F
Age (years), mean (SD)	9.05 (1.5)
Siblings, *n*	2.3 (1.7)
Ethnicity, *n*	
White	24
African American	33
Hispanic	19
BMI z score	1.12 (0.9)
% overweight/obese	57.9%

**Parent**	

Gender	3M, 73 F
% mothers	89%
Age	
< 30 years	16
30-39 years	38
>39 years	22
Ethnicity	
White	24
African American	33
Hispanic	19
Mexican-American	14
Years in US	13.04 (7.4)
Education	
< HS	19
HS	19
>HS	38
Marital status (% married)	50%
BMI	31.88 (8.8)
% overweight, obese	76%

*Note*. Sample sizes (n) are reported unless otherwise noted by the mean (SD) or percent (%);

M = Male, F = Female; BMI = Body mass index

Data was collected from April-June, 2007.

On average children had five valid days of data and wore the accelerometer for 13 hours per day. Table 2 shows that children accumulated, on average, 113.43 ± 37.0 minutes of moderate-vigorous physical activity (MVPA) per day. Almost all of this, however, was moderate activity (MPA) (104.80 ± 32.2 or 93%) with very few minutes accumulated in vigorous (VPA) (6.80 ± 5.6) and very vigorous (VVPA) (1.82 ± 2.8) activity. Mean counts per day were 50.89 (15.5) per 10,000. Boys participated in more MPA and MVPA than girls, but there were no other differences by gender for the other physical activity variables (e.g. VPA). There were no differences in children's physical activity level by weekday versus weekend (data not shown).

**Table 2:** Children's objectively measured physical activity

	**Boys (n = 26)**	**Girls (n = 50)**	**Total (n = 76)**
	
Counts/d^†^	55.60 (13.33)	48.40 (16.15)	50.89 (15.51)
*Minutes engaged in activity:*			
Sedentary	114.35 (15.98)	116.94 (24.63)	116. 17 (21.84)
Light	310.76 (53.02)	314.01 (56.24)	312.98 (54.66)
Moderate (MPA)**	115.11 (29.17)	99.38 (32.76)	104.80 (32.22)
Vigorous (VPA)	8.41 (5.56)	5.94 (5.53)	6.80 (5.63)
Very vigorous (VVPA)	2.03 (2.47)	1.71 (2.96)	1.82 (2.79)
Moderate-vigorous (MVPA)**	125.54 (33.01)	107.03 (37.77)	113.43 (37.00)

*Note. *Mean (SD)

^†^per 10,000

**Significantly different at p < 0.05

An *uninvolved *parenting style was most common (n = 24) followed by *authoritative *(n = 19), *authoritarian *(n = 17) and *permissive *(n = 16). The mean scores for the activity-related parenting practices were 2.79 ± 0.9 for logistical support (range: 1-4), 2.66 ± 0.8 for explicit modelling (range: 1-4), 2.66 ± 0.9 for monitoring (range: 0-4), 1.75 ± 1.1 for discipline (range: 0-4), 2.99 ± 1.1 for reinforcement (range: 0-4), 2.62 ± 1.2 for control (range: 1-5), and 3.48 ± 0.9 for limit setting (range: 1-5).

### Relationship between parenting styles and activity-related parenting practices

The Spearman rank correlation analysis between parenting style typologies and practice subscales showed few significant relationships (Table 3). The majority of effect sizes were small and ranged between 0.24-0.31. An *authoritarian *parenting style was most frequently, and negatively, associated with the *explicit modeling*, *monitoring*, and *discipline *subscales. *Authoritative *and *permissive *parenting styles were associated with parental reinforcement and monitoring, respectively. No associations were noted between an *uninvolved *parenting style and the activity-related parenting practices.

**Table 3:** Association (Spearman correlation) between activity-related parenting practices and parenting style typologies (n = 76).

	***Parenting styles***			
	
	**Authoritative**	**Authoritarian**	**Permissive**	**Uninvolved**
	
***Activity-related parenting practices***				
Logistical support	0.06 (0.60)	-0.05 (0.65)	0.14 (0.24)	-0.13 (0.27)
Explicit modelling	0.17 (0.15)	**-0.31 (0.01)**	0.06 (0.63)	0.07 (0.56)
Monitoring	0.07 (0.53)	**-0.31 (0.01)**	**0.25 (0.03)**	-0.01 (0.94)
Discipline	-0.02 (0.90)	**-0.30 (0.01)**	0.18 (0.12)	0.13 (0.28)
Reinforcement	**0.24 (0.04)**	-0.17 (0.15)	0.13 (0.28)	-0.18 (0.13)
Control	0.13 (0.25)	-0.09 (0.46)	-0.16 (0.18)	0.09 (0.44)
Limit Setting	0.07 (0.54)	-0.05 (0.68)	0.17 (0.15)	-0.17 (0.14)

*Note. Spearman *correlation (r_s_) and p-value (p); **in bold**: *p *< 0 .05 and *p *< 0 .01

### Associations between child physical activity and general parenting styles and activity-related parenting practices

Children of parents with a *permissive *style accumulated more minutes of MVPA than children of parents with an *uninvolved *style (127.5 vs. 97.1, p < 0.05) and an *uninvolved *parenting style was negatively associated with MVPA (r_s _= -0.30, p < 0.01). Parent logistical support was the only activity-related parenting practice associated with child MVPA (r_s _= 0.28, p = 0.01). Parents who provided above average levels of support had children who participated in more minutes of moderate activity (114.2 vs. 98.3 min., p = 0.03) and moderate-vigorous activity (123.1 vs. 106.7 min., p = 0.05), but not vigorous activity.

### Regression models predicting child physical activity

As previously stated, regression models were run using an *authoritarian *style as the referent group. We also conducted alternate analyses to determine whether the significance or direction of association changed when an *authoritarian *style was accounted for in the model. No significant changes were demonstrated and the model with an *authoritarian *style as the referent group proved to be the best fit for the data. All models controlled for child age, gender, and number of siblings -- covariates associated with child physical activity level. In a multiple regression model, only an *uninvolved *parenting style was negatively associated with total counts and minutes of child MVPA while controlling for the covariates (Table 4, Model 1). None of the activity-related parenting practice variables significantly predicted child physical activity while controlling for the same covariates (Table 4, Models 2-8). However, in the moderated multiple regression models testing the role of parenting style on the relationship between activity-related practices and children's physical activity (Table 5) significant interaction terms were noted between a *permissive *parenting style and *monitoring *(Model 9) and a *permissive *parenting style and *reinforcement *(Model 10).

**Table 4:** Association between parenting behavior and child physical activity while controlling for known covariates.

	**Total Counts/d**^**a**^** (per 10,000)**					**Minutes of MVPA**^**b**^				
	
Dependent:	B	SE	Std. β	*t*	*p*	B	SE	Std. β	*t*	*p*
*Parenting style*										
Model 1:										
Authoritative	-5.36	4.86	-0.15	-1.10	0.27	-11.15	11.8	-0.13	-0.95	0.35
Permissive	0.25	4.95	0.01	0.05	0.96	-1.12	12.0	-0.01	-0.09	0.93
**Uninvolved**^**†**^	**-10.19**	**4.48**	**-0.31**	**-2.27**	**0.03**	**-23.38**	**10.9**	**-0.30**	**-2.15**	**0.04**
										
*Parenting practices*										
Model 2:	2.80	1.92	0.16	1.46	0.15	7.34	4.6	0.18	1.60	0.12
Logistical support										
										
Model 3:	-0.50	2.18	-0.03	-0.23	0.82	-2.02	5.2	-0.04	-0.38	0.70
Explicit modeling										
										
Model 4:	-1.44	1.81	-0.09	-0.77	0.44	-5.13	4.4	-0.13	-1.15	0.25
Monitoring										
										
Model 5:	-0.06	1.58	-0.00	-0.04	0.97	-2.23	3.8	-0.66	-0.59	0.56
Discipline										
										
Model 6:	0.02	1.56	0.00	0.01	0.99	-1.57	3.74	-0.05	-0.42	0.68
Reinforcement										
										
Model 7:	-2.04	1.46	-0.16	-1.40	0.17	-4.38	3.51	-0.14	-1.25	0.22
Control										
										
Model 8:	0.06	1.79	0.04	0.34	0.74	0.28	4.30	0.01	0.07	0.95
Limit setting										

Note. *Reference group for parenting style (authoritarian)

^**†**^p < 0.05; All models adjusted for child characteristics (gender, age, number of siblings).

Adjusted R-square: Model 1a = .217; Model 1b = .190; Model 2a = .181; Model 2b = .172; Model 3a = .157; Model 3b = .144; Model 4a = .156; Model 4b = .149; Model 5a = .156; Model 5b = .146; Model 6a = .156; Model 6b = .144; Model 7a = .179; Model 7b = .160; Model 8a = .158; Model 8b = .142

**Table 5:** Moderated regression analysis examining the association between parenting styles and practices with child physical activity.

	**Total Counts/d**^**a**^** (per 10,000)**					**Minutes of MVPA**^**b**^				
Dependent:	B	SE	Std. β	*t*	*p*	B	SE	Std. β	*t*	*p*
Model 9: Parenting Style*Monitoring										
Monitoring	-2.52	3.64	-0.15	-0.69	0.49	-6.39	8.89	-0.16	-0.72	0.48
Authoritative	-4.19	5.18	-0.12	-0.81	0.42	-7.97	12.66	-0.09	-0.63	0.53
Permissive	-3.26	5.55	-0.09	-0.59	0.56	-7.48	13.56	-0.08	-0.55	0.58
Uninvolved	-9.30	4.73	-0.28	-1.96	0.05	-21.20	11.54	-0.27	-1.84	0.07
Authoritative × Monitoring	1.45	5.80	0.03	0.25	0.80	1.25	14.16	0.01	0.09	0.93
**Permissive × Monitoring**^**†**^	**1.38**	**6.10**	**0.32**	**2.25**	**0.03**	28.58	14.91	0.28	1.92	0.06
Uninvolved × Monitoring	-2.92	4.60	-0.11	-0.64	0.53	-7.94	11.24	-0.12	-0.71	0.48
										
Model 10: Parenting Style*Reinforcement										
Reinforcement	0.04	2.37	0.00	0.02	0.98	-0.35	5.76	-0.01	-0.06	0.95
Authoritative	-3.88	4.75	-0.11	-0.82	0.42	-5.17	11.55	-0.06	-0.45	0.66
Permissive	-8.18	5.06	-0.22	-1.61	0.11	-19.66	12.29	-0.22	-1.60	0.12
Uninvolved	-1.15	4.20	-0.35	-2.73	0.01	-26.28	10.20	-0.33	-2.58	0.01
Authoritative × Reinforcement	-4.83	3.95	-0.16	-1.22	0.23	-15.28	9.60	-0.21	-1.59	0.12
**Permissive × Reinforcement**^**†**^	**1.82**	**5.32**	**0.41**	**3.43**	**0.00**	**40.54**	**12.93**	**0.38**	**3.14**	**0.00**
Uninvolved × Reinforcement	-3.04	3.48	-0.12	-0.87	0.39	-8.23	8.46	-0.13	-0.98	0.33

*Note. *Reference group for parenting style (authoritarian)

^**†**^p < .05; All models adjusted for child characteristics (gender, age, number of siblings).

Model 9a: R^2 ^change = .247, F(4,70) = 4.202, p = .004; R^2 ^= .372, Adj R^2 ^= .274; Model 9b: R^2 ^change = .220, F(4,70) = 3.553, p = .004; R^2 ^= .340, Adj R^2 ^= .237;

Model 10a: R^2 ^change = .317, F(4,71) = 6.075, p = .000; R^2 ^= .435, Adj R^2 ^= .348; Model 10b: R^2 ^change = .307, F(4,71) = 5.67, p = .000; R^2 ^= .413, Adj R^2 ^= .323

**Figure 2:**
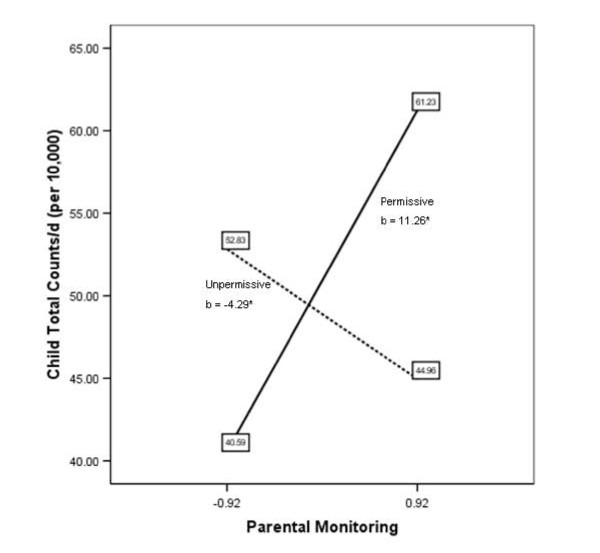
**Regression lines for parent monitoring and counts of child physical activity, moderated by parenting style**. For the *permissive *parenting style group, findings suggest that higher levels of monitoring are associated with accumulation of more physical activity counts (per 10,000) by the child (*b *[unstandardized regression coefficient] = 11.26, p = 0.02). Whereas higher levels of monitoring were associated with slightly lower accumulation of child physical activity counts (*b *[unstandardized regression coefficient] = -4.29, p = 0.03) for the *unpermissive *parenting style group.

**Figure 3:**
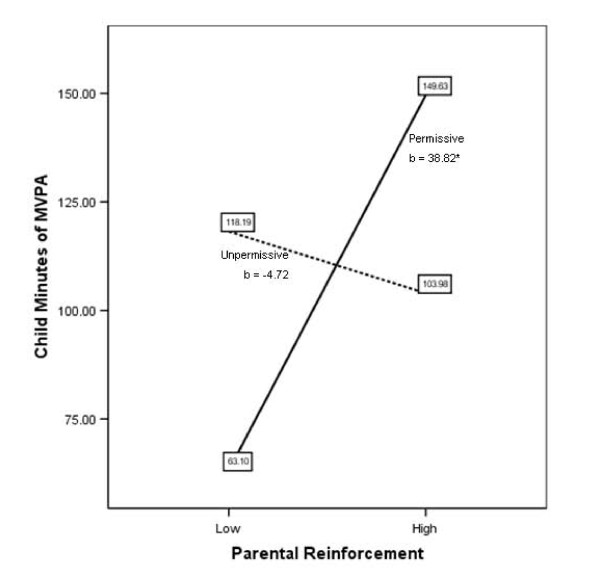
**Regression lines for parent reinforcement and minutes of  child MVPA, moderated by parenting style**. For the *permissive *parenting style group, findings suggest that higher levels of reinforcement are associated with accumulation of more minutes of MVPA by the child (*b *[unstandardized regression coefficient] = 38.82, p = 0.01). Note. MVPA = moderate-vigorous physical activity.

A post-hoc analysis to probe for the significant moderation effects were conducted [49]. Considering the dichotomization of the *permissive *parenting style variable where a value of 1 represents the presence of a *permissive *parenting style and a value of 0 reflects the absence of a *permissive *parenting style, we have changed the terminology. An "*unpermissive*" parenting style refers to the absence (or opposite) of a *permissive *parenting style (Figure 2). Figure 2 suggests that for the *permissive *parenting style group, higher parental *monitoring *of child physical activity was associated with greater accumulation of total counts by the child (*b *= 11.26 per 10,000, p = 0.02). In contrast, for the *unpermissive *parenting style group findings suggest a small, but inverse relationship between low and high monitoring practices and counts of child physical activity (*b *= -4.29 per 10,000, p = 0.03). Figure 3 illustrates the significant permissive*reinforcement interaction terms and suggests that only for the *permissive *parenting style group higher levels of *reinforcement *are associated with more minutes of child MVPA (*b *= 38.82, p = 0.01). The post-hoc probing of the significant permissive*reinforcement interaction for total counts of child activity showed the same relationships as Figure 3.

## Discussion

This paper describes the parenting styles and activity-related practices of a group of US families living in under-resourced rural communities. One of the primary goals of this study was to assess whether parenting styles and practices were associated with child physical activity. Our findings suggest that both parenting style and certain parenting practices are significantly associated with child physical activity. This is consistent with the limited amount of research in this area [9,17,26,50] and also extends this work by documenting other aspects of parenting style (*permissive *and *uninvolved*) that have not been previously investigated with respect to child physical activity. In this study, *permissive *parenting style was associated with the most minutes of child physical activity and *uninvolved *parenting style the least. An *authoritative *parenting style was not associated with child physical activity in this study. Our findings may be in contrast to other studies [26] because other studies have not measured parenting style in a comprehensive or systematic way (e.g. studies that have focused solely on authoritative/authoritarian typologies). Given that rural children living in under-resourced communities have limited access to physical activity opportunities, parents may need to provide a high level of support and encouragement for their child to be active and also place few restrictions or demands on their children's time (characteristic behaviors of a *permissive *parenting style). This may also be supported by the finding that providing children with logistical support also resulted in more minutes of MVPA for children.

To our knowledge, no study has examined the relationship between specific activity-related parenting practices and parenting style. Similar work in other disciplines has demonstrated a link between feeding practices and parenting style [28,46]. For instance, working with a different sample of rural parent-child dyads, Hubbs-Tait et al., [28] found that restriction and pressure to eat were negatively correlated to *authoritarian *and *permissive *parenting styles. However, Hughes and colleagues [46] found that *authoritarian *parents were more likely to put pressure on their children to eat, *permissive *parents were less likely to use restriction, and *authoritative *parents were more likely to monitor. We were surprised to find few relationships between parent's activity-related practices and their general parenting style. As expected, praising a child for his/her physical activity habits (reinforcement) was positively associated with an *authoritative *style, but contrary to our expectations, both discipline and monitoring of child physical activity were negatively associated with an *authoritarian *parenting style.

There may be several potential reasons why we found few relationships between parenting style and activity-related parenting practices. First, the sample size was small. Although sufficient to run these analyses, the loss of accelerometer data was slightly higher than expected and additional studies with larger sample sizes are warranted. Second, these findings suggest that more work is needed to understand the goals and values parents place on their child's physical activity that may differ from their general views of parenting. For instance, a recent study found that parents of 5-7 year old children in Spain valued socially acceptable behaviour over their child being active, which led them to have a more relaxed attitude toward their child's activity [51]. Lastly, the control, reinforcement, discipline and monitoring subscales were measured by one or two items and may therefore not adequately represent the construct they attempt to measure. Although the authors of the PEAS instrument contend that previous research has found that two items may sufficiently identify a factor, future research should develop additional items to assess these constructs as they relate to child physical activity habits [18].

It is also unclear why parenting practices other than logistical support were not independently associated with their child's physical activity. In a longitudinal study of adolescents, Ornelas and colleagues [27] did not find an association between parental monitoring and physical activity despite their hypothesis that this type of parenting strategy -if either too directive or restrictive - may negatively impact children's physical activity. As mentioned previously, more work is also needed to adequately measure the constructs related to parental discipline, control, monitoring, and reinforcement of their child's physical activity habits. However, findings from this study suggest that parenting style may in fact moderate the relationship between parent's activity related practices and their child's physical activity. For instance, monitoring and reinforcement were only associated with child physical activity when expressed in the context of a *permissive *parenting style. This provides some validation to the rationalization by Ornelas [27] that certain practices may be beneficial, but only when the emotional context within which they are expressed to the child is balanced appropriately. Overt support and encouragement of healthy behaviors is likely to be important in children's adoption of health behaviors such as physical activity, however, parents need to be aware of the emotional context employed. Davison and Campbell [14] theorize that children are responsive to parents' moral support of their physical activity (e.g. watching them compete in athletic events) and the general feeling that physical activity is valued in the family, yet may resent physical activity and consider it a chore when it is regimented or promoted as a method of weight loss. This may be one possible explanation for the finding in this study that the relationship between children's accumulation of total counts of activity and parental monitoring was negative when in the presence of an *unpermissive *parenting style.

Additionally, it has been suggested that parents may take a different approach to their parenting strategies toward physical activity among children who are overweight than toward those who are not [52]. In this study, child BMI z score was not related to any physical activity variable. Since the majority of children's active time was spent in moderate, not vigorous activity, further investigation is warranted regarding the intensity of rural children's physical activity and parent's perception of their child's activity level. A large proportion of children in this sample were either overweight or obese, which is consistent with other literature suggesting that for rural children, overweight prevalence is higher than in urban areas [4,53,54]. These findings may suggest that the level of energy expenditure by children in this study has been insufficient to prevent excess weight gain. More work is needed to understand why children living in rural, under-resourced areas do not engage in more vigorous activity, especially if this level of activity is necessary to address the growing prevalence of overweight among children [55-57].

It is important to evaluate these findings in the context of the study limitations. The small sample size would not support certain subgroup analyses (e.g. separating mothers vs. fathers or girls vs. boys). The sample also represents a diverse group of parent-child dyads, yet generalizability may still be limited given that the sample is predominantly mother-daughter dyads, the focus is on families living in under-resourced rural areas, a lack of representation from other minority groups, and the randomly selected, but convenient sample, from which the participants were recruited. Findings may be specific to rural areas, which despite its characteristic open space often has fewer resources for children to be active as compared to urban environments [58,59]. Since this was a cross-sectional study, one cannot discriminate the direction of influence. Parents are not parenting in isolation, but in response to several factors including child traits [60]. Thus, the parent-child relationship is bi-directional and additional studies are needed to understand causal pathways between parenting behaviors and child physical activity that include measures of specific child behaviors (e.g. temperament) or potential mediating variables (e.g. self-efficacy).

Overall, this work suggests that moderating factors such as parenting style should be considered in future studies of parental influences over child physical activity levels. In general, a more focused investigation of theoretical or methodological moderators in addition to mediators and confounders may clarify previously indeterminate findings across investigations [7,61]. For instance, parental reinforcement of child physical activity may be moderated by parenting style, but may be mediated by children's efficacy and competency [7]. It would also be interesting to include measures of child report of parental behavior to determine the similarities and differences with parental report of their own behavior. How children perceive their parents - as supportive or unsupportive - may be an important factor and whether differences exist for parent-child gender pairs. Other factors such as family cohesion, parental engagement, and parent-child communication have been evaluated in a few studies but more work is needed [27]. Additionally there may be other domains of parental support that have not yet been evaluated in the literature (e.g. the extent to which parents alter the home environment to be supportive of child physical activity) [62] and whether parental influence may have an impact on weekday versus weekend activity levels for children.

Currently, interventions have demonstrated mixed results and the effectiveness of family involvement methods or parental components in promoting physical activity in children remain unclear [63]. One reason may be that interventions have not accounted for certain factors - such as parenting style - which may alter the effectiveness of the parenting practices targeted in the intervention [64]. Development of theory-specified, empirically verified models that predict family and parent influences on children's physical activity behaviors are needed to develop effective interventions.

## Conclusions

Overall, this work extends the current literature by demonstrating the potential moderating role of parenting style on the relationship between activity-related parenting practices and children's objectively measured physical activity while controlling for known covariates. Our findings add to the growing evidence base, which suggests that parents need to be aware of their behaviors that may or may not have unintended consequences on their child's health [65]. Future studies in this area are warranted to identify the mechanism by which parent's influence their child's physical activity behavior.

## Competing interests

The authors declare that they have no competing interests.

## Authors' contributions

EH, SOH, JPG, RRH, CDE made substantial contributions to the conception, design, acquisition, analysis and interpretation of the data. EH, CDE have been involved in drafting the manuscript or revising it critically for important intellectual content. EH, SOH, JPG, RRH, CDE have given final approval of the version to be published. At the time this study was conducted, the first author (EH) was a Doctoral Candidate at the John Hancock Research Center on Physical Activity, Nutrition, and Obesity Prevention, Tufts University Friedman School of Nutrition Science and Policy, Boston, MA, USA. EH is now a Cancer Prevention Fellow at the National Cancer Institute, National Institutes of Health, Bethesda, MD, USA.

## Funding disclosure

This research was supported by awards from the Centers for Disease Control and Prevention (Public Health Dissertation Research Award, Grant 1R36DP001325-01) and New Balance Foundation with additional funding provided by Save the Children, US Programs and the Robert Wood Johnson Foundation (Grant 59458).
